# Behavioral, Physiological and EEG Activities Associated with Conditioned Fear as Sensors for Fear and Anxiety †

**DOI:** 10.3390/s20236751

**Published:** 2020-11-26

**Authors:** Jui-Hong Chien, Luana Colloca, Anna Korzeniewska, Timothy J. Meeker, O. Joe Bienvenu, Mark I. Saffer, Fred A. Lenz

**Affiliations:** 1 Department of Neurosurgery, Johns Hopkins University, Baltimore, MD 21287-7713, USA; jchien7@jhmi.edu (J.-H.C.); tmeeker3@jhmi.edu (T.J.M.); msaffer3@jhmi.edu (M.I.S.); 2 Department of Pain Translational Symptom Science, School of Nursing, University of Maryland, Baltimore, MD 21201-1595, USA; colloca@umaryland.edu; 3 Department of Anesthesiology, School of Medicine, University of Maryland, Baltimore, MD 21201-1595, USA; 4 Department of Neurology, Johns Hopkins University, Baltimore, MD 21287-7713, USA; akorzen@jhmi.edu; 5 Department of Psychiatry and Behavioral Sciences, Johns Hopkins University, Baltimore, MD 21287-7713, USA; obienve1@jhmi.edu

**Keywords:** scalp EEG, Event Related Spectral Perturbation, Event Related Potential, fear conditioning, human, expectation, fear, anxiety

## Abstract

Anxiety disorders impose substantial costs upon public health and productivity in the USA and worldwide. At present, these conditions are quantified by self-report questionnaires that only apply to behaviors that are accessible to consciousness, or by the timing of responses to fear- and anxiety-related words that are indirect since they do not produce fear, e.g., Dot Probe Test and emotional Stroop. We now review the conditioned responses (CRs) to fear produced by a neutral stimulus (conditioned stimulus CS+) when it cues a painful laser unconditioned stimulus (US). These CRs include autonomic (Skin Conductance Response) and ratings of the CS+ unpleasantness, ability to command attention, and the recognition of the association of CS+ with US (expectancy). These CRs are directly related to fear, and some measure behaviors that are minimally accessible to consciousness e.g., economic scales. Fear-related CRs include non-phase-locked phase changes in oscillatory EEG power defined by frequency and time post-stimulus over baseline, and changes in phase-locked visual and laser evoked responses both of which include late potentials reflecting attention or expectancy, like the P300, or contingent negative variation. Increases (ERS) and decreases (ERD) in oscillatory power post-stimulus may be generalizable given their consistency across healthy subjects. ERS and ERD are related to the ratings above as well as to anxious personalities and clinical anxiety and can resolve activity over short time intervals like those for some moods and emotions. These results could be incorporated into an objective instrumented test that measures EEG and CRs of autonomic activity and psychological ratings related to conditioned fear, some of which are subliminal. As in the case of instrumented tests of vigilance, these results could be useful for the direct, objective measurement of multiple aspects of the risk, diagnosis, and monitoring of therapies for anxiety disorders and anxious personalities.

## 1. Introduction

Anxiety disorders and related personality traits (especially neuroticism and trait anxiety) have substantial effects on the public health as the most common mental disorders in the USA and worldwide [1,2,3] Anxiety disorders are disabling conditions, which cause significant suffering, and incur significant disability and costs [4,5,6], since they tend to have early onsets, chronic courses, and affect 29% of the population across the lifespan (18% during any year) [7,8]. Anxiety disorders may be particularly distressing because they can occur in the face of relatively intact physical and intellectual functioning, and may increase the symptom burden of medical diseases [9,10] Post-traumatic stress disorder in soldiers and civilians has historically been categorized with the anxiety disorders and affects 7% of the US population during their lifetime (3.5% during any year) [9,10]. Anxiety-related personality traits are similarly burdensome, increasing the risks for divorce, unemployment, and disability, while affecting the incidence, prevalence, and comorbidity of common mental disorders, and impairing functioning, symptom remission, and recovery from these disorders [11]. 

**Figure 1:**
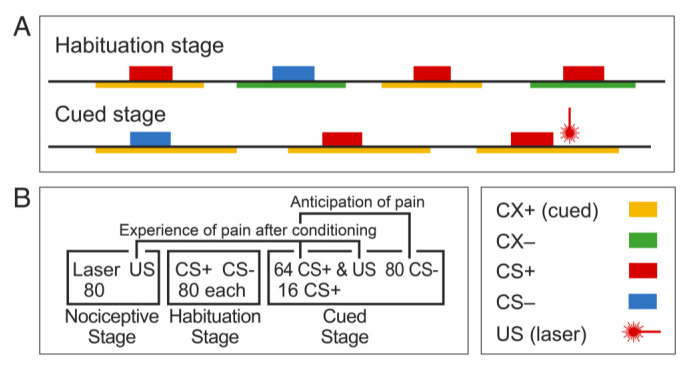
Aversive Conditioning Protocol: (**A**) Cartoon of stages including the cued stage composed of a train of visual stimuli in which an unconditioned stimulus (US—painful laser pulse) is paired with one conditioned light stimulus in 75% of trials (CS+, image of a lamp lighted red signaling threat—80 total (see Panel B), while the second light is not paired (CS−, lamp lighted blue signaling safety—80), and the CS+ and CS− are presented randomly in the cued context (CX+, yellow underline, image of an office with lamp as above). CX, green underline is the extinction context, not reported here. After pairing of stimuli, the CS+ elicits a conditioned response (CR) including the Skin Conductance Response (SCR) and the ratings described below. The protocol is randomized for the (i) order of CSs and pairing of CS+ and US (ii) duration (within limits) of intertrial intervals, CSs, CXs, and CX onset to onset of CS. (**B**) Each stage has 2 blocks. The protocol begins with 2 blocks in the Nociceptive Stage (total of 80 stimuli) followed by the Habituation stage (160 CSs total). Ratings are performed in all intervals between blocks, and after the protocol. Anticipation is measured by the contrast between CS+ vs. CS− in the Cued Stage; experience of cued pain is measured by the contrast of US between the Cued vs. the Nociceptive Stage, and the US skin conductance response (SCR) following CS+ vs. CS− in the cued stage. This Figure is adapted from [12].

There are a number of existing metrics for measuring fear or anxiety in patients and subjects including the Anxiety Sensitivity Index (ASI) [13,14], the State-Trait Anxiety Inventory (STAI) [15], the Hospital Anxiety and Depression Scale [16], the Hamilton Anxiety Rating Scale [17], the Fear of Pain Questionnaire [18], the Fear Avoidance Beliefs Questionnaire [19], the Waddell Beliefs Questionnaire [20], the Pain Catastrophizing Scale [21], and the Pain Anxiety Sensitivity Scale [22,23]. In fear conditioning protocols, conditioned responses (CRs, Legend Figure 1) include physiological metrics and rating scales for psychological domains of fear and anxiety [12,24,25], and are directly related to fear and anxiety [26,27,28,29,30]. In these protocols, self-report rating scales can be used for two separate domains of fear or expectation of the laser, as characterized by anticipation and experience of cued pain. 

**Figure 2:**
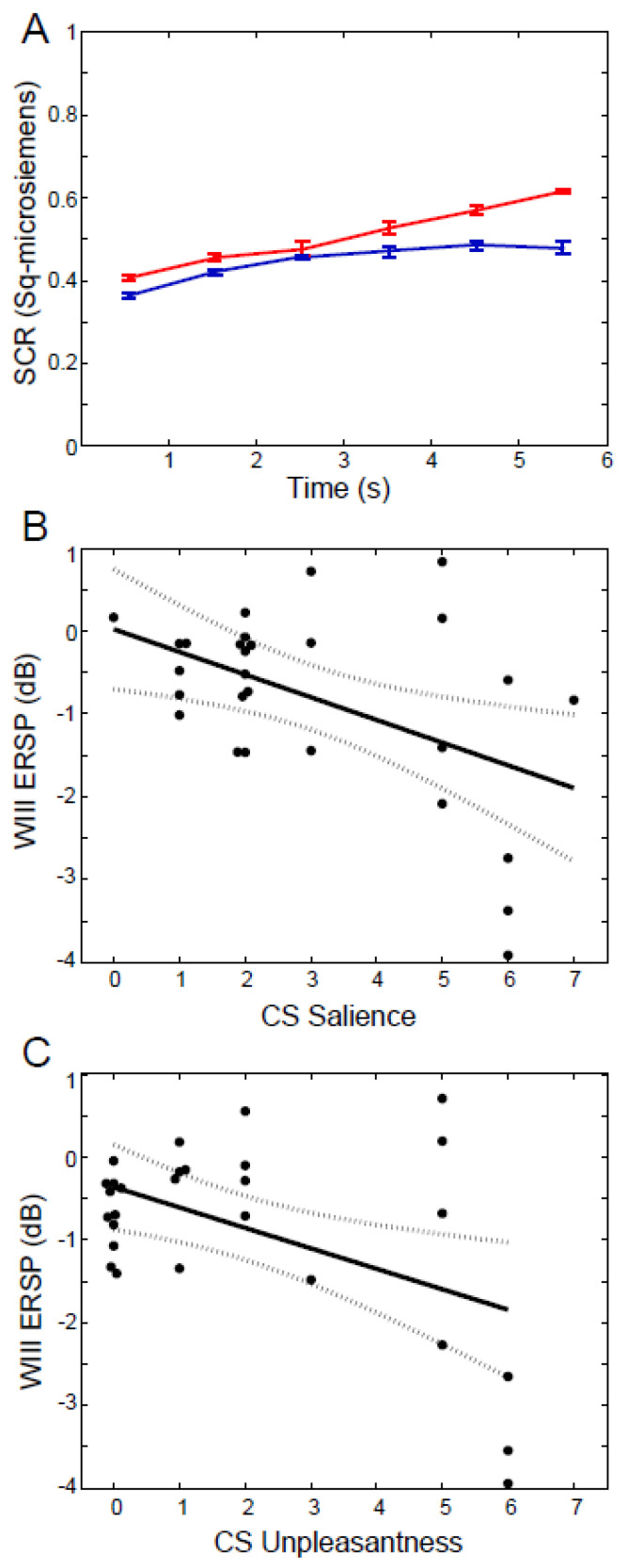
Skin conductance response (SCR) and CS Ratings during the Acquisition stage. (**A**) SCR ((mean +/− SEM)) at intervals after the CS+ (red line) and CS− (blue line). (**B**,**C**) are linear regression models (solid line) plus the 5% and 95% confidence bounds, dotted lines of WIII ERD vs. CS Salience and Valence across Subjects. See Text. Adapted from [12].

The first of these domains is measured by the response to the conditioned stimulus (CS+—anticipation of pain) and includes CRs of skin conductance response (CS SCR), as well as ratings of the likelihood of an association of CS+ with the laser (expectancy), and the ability of a stimulus to command attention (salience) or to be described as unpleasant (valence) (Figure 2) [24,25] Other metrics include rating scales for anxiety [31,32] and avoidance [33] Some of these scales can be carried out without the subject being fully aware of the assessment, such as self-report Manikin [34] or economic scales [35,36]. These metrics will be bolstered by comparison with physiological metrics such as SCR and EEG of which the subjects have no awareness. 

There are hundreds of studies of both EEG and fMRI modalities suggesting sensors that could become biomarkers for fear and anxiety without the subjects’ full awareness [37,38,39]. A detailed review of these modalities is beyond the scope of this manuscript, which instead focuses upon the results obtained with our novel protocol incorporating stimuli and CRs related to the two domains in the human literature of expectancy i.e., fear conditioning and anticipation of pain. We do, however, present short summaries of the results of imaging, EEG and magneto-encephalographic based research related to fear and anxiety. We first consider EEG and fMRI techniques, and then describe behavioral and physiological sensors during conditioned fear that could be developed into biomarkers for fear and anxiety. 

EEG metrics have included spontaneous EEG, stimulus evoked potentials (phase-locked, ERP, see Abbreviations) and spectral activity following an event such as a stimulus (non-phase-locked e.g., ERS). Spontaneous EEG activity in some anxiety disorders shows increased delta/theta and decreased alpha, beta, and gamma bands [40,41,42], and more frequent transitions between these frequency bands. These changes are neither specific nor selective for anxiety disorders but are found in OCD, ADHD, and schizophrenia but not in PTSD and may be related to cognitive function in the latter two diagnoses [43,44]. Evoked late ERP waves include the P300 and mismatch negativity may be related to cognitive processes such as attention and expectancy, and be increased in patients with PTSD vs. trauma-exposed controls during fear conditioning [45,46,47]. Finally, ERSP and connectivity based upon scalp EEG activity was related to increased salience and valence of the conditioning stimuli in patients with PTSD [12,48,49].

**Abbreviations: Conditioning:** e.g., CS, US, CR, SCR see Figure 1 Legend, CR conditioned response, includes SCR, and ratings of valence, salience, CS+ laser association. 

**Electrical signals: LFP (or EcoG)** Local Field Potential is the electrical activity of multiple neurons recorded directly from the brain while **EEG** refers to refers to scalp recordings by 10–20 array (HH Jasper). 

**Frequency Bands:** delta 1-3 Hz, theta 4–7, alpha 8–12, beta 2–30, low gamma 30–70. A range of frequency and time post-event ERSP define **Windows** in ERSP time frequency plots.

**Activations—ERSP** Event Related Spectral Perturbation (in DB) is post stimulus oscillatory power over baseline which can: increase (**ERS** > 1 hot colours in Time Frequency plots of ERSP) or decrease (**ERD** < 1, cold colours).

**ERP:** Event Related Potentials evoked by a stimulus (Figure 2A,B): Laser (**LEP**) composed of N2- early negative peak, P2- later positive peak, LP- Late Potential, and Visual (**VEP**) P300 attention-related late positivity. BOLD hemodynamic fMRI variable often reflecting the difference between events like the CS+ and CS−.

**Anatomy AMY** amygdala, **HIPP** hippocampus. 

Magneto-encephalographic fields and ERSP (gamma band) and the location their generators have been related to fearful vs. neutral faces as a function of the likelihood a threatening stimulus will occur, and of awareness vs. masking of the stimulus [50]. In the former, healthy subjects and patients with anxiety disorders showed increased activity in the dorsolateral prefrontal cortex when the threat was not cued [51]. Patients with panic disorder and specific phobia showed long lasting activation over anterior inferior cingulate with or without the cue that was correlated with ratings of psychological activation and anxiety sensitivity [51]. Patients with specific phobia and panic disorder both showed altered magneto-encephalic activity over associational (parietal) cortex. Another study found that conscious awareness was associated with widespread cortical gamma band synchrony activity following neutral faces, while fearful faces were associated with cortical and amygdala (AMY activations [50]. These studies suggest that EEG and magneto-encephalographic spectral power, evoked responses, psychological ratings during presentation of fearful stimuli, and fear conditioning protocols may be sensors for fear and anxiety. A meta-analysis reviewing 97 studies of magneto-encephalographic activity in patients with social anxiety disorder found that the fusion of magneto-encephalographic, EEG, and fMRI results was more effective than EEG alone [39]. These studies suggest that electrophysiological activities are related to fearful stimuli and conditioned fear and may be sensors for increased trait anxiety and anxiety disorders.

fMRI is another modality that has also been explored as an approach to the development of biomarkers. In comparison to electrical and magnetic signals, fMRI has lower temporal resolution, is an indirect measure of neural activity, and is based upon assumptions that are still debated [52,53,54,55]. fMRI is subject to artifacts such as those related to venous flow adjacent to the amygdala and signal loss near sinuses [56]. Therefore, electrical signals, particularly those recorded directly from the brain, are the gold standard for assaying neural activity [29]. Sensors based on structural MRI like cortical thickness are useful to measure trait anxiety or risk of developing anxiety disorders, but do not capture the dynamics of changes in state of emotion or mood that can change rapidly [57,58,59]. On the other hand, MRI has the advantage of surveying the whole brain, while scalp EEG is the sum of activity at multiple structures at low spatial resolution. 

Early meta-analyses commonly found fMRI found changes in activity in the AMY and components of the cingulate cortex, particularly anterior, dorsal anterior, or ventral medial cingulate [60,61], although segmentation of the cingulate could confound the interpretation of fMRI results [62,63]. Within diagnoses, the pattern of fMRI signals was often very different between similar studies. Two studies found AMY vs. cingulum (anterior and dorsal anterior) activations during a fearful faces protocols as a way to identify sensors for the effect of treatment with the serotonin specific reuptake inhibitor venlafaxine [64,65,66]. The activations in these studies showed double dissociation so that the results were not just inconsistent but opposite. More recent meta-analyses of all anxiety disorders, including PTSD, and trait anxiety concluded that patterns of fMRI regions of interest were not characteristic features of individual anxiety disorders [67]. The range of results in these studies may have been complicated by the range of diagnoses, imaging techniques, scanning parameters, and regions of interest, in addition to the technical issues described above.

There are some cases in which the efficacy of a therapy may be related to the activation of a particular region across diagnoses by the same protocol although there are differences in the pattern of fMRI activity by each diagnosis. The BOLD signal that is the sensor may then be based upon the response during either a behavioral protocol or administration of a drug [68,69]. Our premise is that behavioral and EEG metrics related to conditioned and unconditioned stimuli in a fear conditioning protocol are sensors that could be developed into biomarkers for fear and anxiety.

## 2. Methods and Rationales

Our methods merge human studies of two common approaches to conditioned fear, one based upon anticipation produced by the conditioning stimuli [70,71,72]. The other is related to the experience of pain and focuses upon the sensory, cognitive, and emotional dimensions of the painful US stimuli [73,74]. Studies of conditioned fear have rarely measured behavioral characteristics of the US while studies of the anticipation of pain have rarely measured CRs of the CSs conditioning by the cued laser association and the CS+ SCR.

We routinely use a painful laser pulse as US in a fear conditioning protocol that has been shown to produce behavioral changes and physiological changes in the central nervous system (Figure 1 and Figure 2), as have other painful stimuli [29,75,76,77,78]. The protocol consists of several stages including: (1) the nociceptive stage, which is similar to conditioning protocols in which the subject is exposed to the US during adjustment of its intensity [72,78,79]; (2) the visual stage, consisting of the CS+ and CS− in either Cx presented in random orders and intervals in one train; and (3) the cued stage that has the pairing of the CS+ and US, which produces CRs. 

The CRs are categorized into those in the domain for the anticipation of pain that is calculated by the CS+ vs. CS− during the cued stage. CRs for the experience include three contrasts (i) the pain of the laser during: the cued vs. the nociceptive stage [24,29,80,81], (ii) the contrast of the US SCR for CS+ events with pairing (75%) vs. those without during the cued stage [12,82,83,84], and (iii) the correlation of CS SCR vs. US pain during the cued stage. 

The CRs are categorized into those in the domain for the anticipation of pain, which is calculated by the CS+ vs. CS− during the cued stage. CRs for the experience include three contrasts: (i) the pain of the laser during: the cued vs. the nociceptive stage [24,29,80,81], (ii) the contrast of the US SCR for CS+ events with pairing (75%) vs. those without during the cued stage [12,82,83,84], and (iii) the correlation of CS SCR vs. US pain during the cued stage. Together, the application of these two techniques give us a novel perspective on both aspects of the aversive conditioning that might be the basis for an objective instrumented test for measurement of fear and anxiety. 

**Figure 3:**
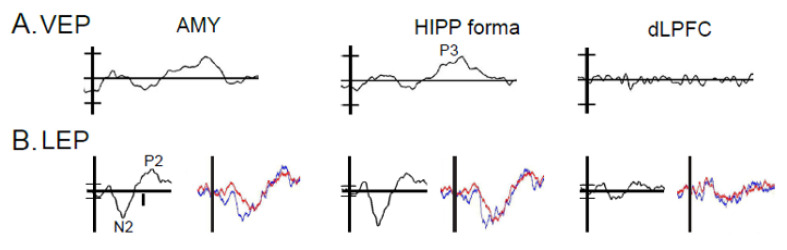
Activations (ERP see Abbreviations) during visual and nociceptive stages for AMY, HIPP, and dlPFC (dorsolateral prefrontal cortex). (**A**) Visual Evoked Responses (VER) where Baseline +/− 3SD is indicated by short horizontal bars on the vertical axis. P3 corresponds to P300 here and is related to the novelty of infrequent stimuli. Horizontal axes (**A**,**B**) −30 to 600 ms. Vertical bar 10 µV, 5 µV in (**A**). (**B**) LEPs shown in black traces. Red and blue traces (right) demonstrate LEPs averaged from separate series of the stimuli included in the black trace and show reproducibility. Vertical bar 10 µV, 5 µV in A.

The studies of the neuroscience of expectation largely report CS+ BOLD signal increases in the amygdala during the anticipation of non-painful USs with measurement of SCR. The studies of conditioned fear report changes in cortical BOLD more frequently than in AMY, and SCR or ratings of the CS or US are often not measured [73] (cf [81]). We have used an aversive conditioning protocol with measurement of physiologic activity and ratings (Figure 1 and Figure 3) to study both *domains* with the painful laser US [85,86]. The ratings of psychological effects in our protocol are particularly valuable because they can only be applied in humans. However, the presentation of multiple rating scales at the same time may lead subjects to make similar responses on all scales [87,88]. Therefore, we have adopted scales with different formats and types such as Visual Analog Scales (VAS), Numerical Rating, Verbal Descriptor Category, and Manikin (psychological activation) Scales [34]. The CS+ laser association is rated by a normal category scale in which the subject may not be aware of the association [35,36].

## 3. Methods: Behavioral Protocol and Metrics

Our fear conditioning protocol includes ratings of all stimuli for pain, conditioning, salience, and confidence in rating performance [12,89,90,91], but does not compromise conditioning [12,90,91]. These ratings in humans may clarify pain-related cognitive and emotional effects of the expectation upon acute pain, chronic pain, and fatigue syndromes such as fibromyalgia [92,93,94]. These ratings and physiological metrics such as, SCR and EEG, may be used as sensors in an objective instrumented test for fear and anxiety in health and disease.

The domain of experience of cued pain is produced by the US and leads to CRs of US SCR and pain that are related to CS SCR but not CS salience or valence (unpublished results). These findings support the proposal of two separate domains of conditioned fear. The anticipation domain may result in fear and avoidance of pain, whereas the experience domain may facilitate the escape from pain, two aspects of the fear of pain [93,95,96]. Our protocol also controls for contexts to enable ongoing studies of extinction, reversal of extinction, and reconsolidation stages that are not reviewed here but which may lead to biomarkers for the response to Cognitive Behavioral Therapy [97,98]. 

In our case, experience is produced by the US and leads to the CRs of US SCR and US-evoked pain, which satisfy criteria for reliability, objectivity, and item homogeneity [99,100]. The US CSRs are related to CS SCR, but not CS salience or valence, which supports the proposal of two separate domains of conditioned fear. Behavioral and EEG measures of anticipation and experience can be both direct and objective, unlike questionnaires and ratings including those for which the subject is unaware of the rating. Sensors based on EEG techniques have some advantages over fMRI-based sensors, which are indirect measures of expectation. fMRI is high-tech and requires expensive experts and equipment plus bricks and mortar that are not found in the great majority of hospitals worldwide, whereas EEG is widely available [101,102]. 

The independence of the ratings for anticipation vs. experience is enhanced by using different scale types and formats. In particular, we use scales with different formats, such as Visual Analog Scales (VAS), Numerical Rating Scale (NRS), Verbal Descriptor Category, and Manikin (psychological activation) Scales for psychological activation [34]. In addition, the CS+ laser association measures the domain of anticipation, which is the likelihood that the CS+ is paired with the laser pulse, and is rated by a monetary normal category scale in which the subject may not be aware of the purpose of the rating. This latter scale is anchored by the question: “If a dollar is a sure thing that one of the lights is associated with the laser, how much would you bet that these two are associated?” [35,36]. The other domain is experience of cued pain, which can produce a nocebo effect—the CR for expectation of an aversive event [84,93,95,96]. 

### 3.1. Methods: Laser, SCR, and EEG

In our studies, the laser stimuli (US, Figure 1) were delivered using a Thulium YAG laser (Themis, StarMedTech, wavelength 2 µm, beam diameter 6 mm, duration 1 ms). The laser stimuli were applied on the dorsum of the right hand. The laser was moved to a slightly different location for each pulse to avoid fatigue or sensitization of nociceptors. The laser energy level for the US was set by a procedure in which levels were increased stepwise from 200 mJ until a pain intensity rating of 4–6 out of 10 was reported, leading to an average laser energy level across subjects of 654 ± 50 mJ. At the end of each block, the participant rated psychophysical metrics of both CSs, and the laser US on an 11-point numerical scale from 0 to 10. For example, US unpleasantness was rated from 0 for the absence of unpleasantness and 10 for the most unpleasant sensation imaginable. 

In our studies, CRs included SCR and ratings of psychological metrics related to CSs, which are described in the introduction. SCR was measured throughout by an isolated skin conductance coupler (Model V71-23, Coulbourn Instruments) with three electrodes on the ventral distal phalanges of the index, middle, and ring fingers. The coupler delivered low distortion sine wave excitation voltage at 100 Hz across the skin and measured the resulting current flow as SCR. 

We report scalp EEG signals recorded using a high density scalp EEG technique with 128 electrodes placed on the scalp with a reference of linked earlobes (Figure 3) [103]. Intracranial EEG was recorded in patients with implanted electrodes for seven to ten days prior to resections for the treatment of epilepsy [104]. Scalp and intracranial EEG signals were amplified and digitized at the sampling rate of 1000 Hz (NeuroPort, Blackrock Microsystems). The event related spectral perturbation (ERSP) was used to estimate the event-related non phase-locked responses induced by the CSs or US [12,105]. ERSP measures significant event-related changes in the power spectrum across different frequencies in the post-stimulus interval by dividing post stimulus spectral estimate by the mean baseline power spectrum (200 ms prior to the stimulus). If an ERSP at a specific frequency and time in the plots shown in figures was larger than 1, that ERSP was an ERS (Event Related Synchronization); if smaller than 1, it was an ERD (Event Related Desynchronization) [106]. 

EEG recordings were re-referenced to an average reference and filtered; all EEG epochs were visually inspected by two independent individuals for artifact rejections, and later by an independent component analysis (EEGlab) [107]. The power spectrum was estimated using FFTs with a Hanning window. Prior to the ERSP analysis, the event-related potentials (ERPs) were estimated by averaging signals for each subject across trials, channels, and behaviors, and ERPs were subtracted from EEG signals related to each stimulus. The thresholds for significance of the post stimulus ERSP estimate were determined by a randomization (bootstrap) procedure analysis based upon spectral estimates of the baseline period i.e., 200 ms prior to the stimulus [12]. 

### 3.2. Results and Interpretation

In addition to behavioral and rating measures, EEG and SCR recordings function as physiological metrics during fear conditioning protocols that may lead to the development of biomarkers for fear and anxiety disorders. Fear conditioning protocols require that the US be aversive, so that human fear conditioning will result from protocols using aversive stimuli including auditory [108], olfactory [109], visual [110,111], painful heat [81,112], or painful cutaneous laser modalities [29,80]. Electric shock is commonly used as a US and have been described as painful [29,75,76,77,78], “highly annoying but not painful,” “mild,” and “intense, but non painful” [72,98,113,114]. Our studies have demonstrated that the laser US is as effective as the electric shock so that results with laser may be generalized to fear conditioning with multiple other aversive stimuli [12,72,115]. 

Anticipation leads to avoidance behaviors related to stimuli associated with a painful injury that might occur again [93,116]. It is also associated with increased fear [117], fewer CS+-US pairings, increased catastrophizing, and neuroticism [12,117,118,119]. Experience leads to behaviors of escape from current pain, like noncompliance with physical therapy that can cause pain [120,121], and to increases in anxiety [12,122]. Therefore, these domains are related to complex effects, and to coping strategies, such as physical inactivity, which predicts worse outcomes in chronic pain from osteoarthritis [123,124] and in chronic “fatigue syndromes” like fibromyalgia [125,126]. The psychological ratings and physiologic measures described above may be sensors that could be used to develop biomarkers for fear and anxiety, while EEG and SCR recordings are physiological measures during fear conditioning and may be sensors for fear and anxiety.

### 3.3. Results: Anatomic Convergence of VEPs and LEPs by Electrode

For either of the behavioral domains to be expressed, there must be convergence of signals at brain components which encode the CS+ and US. Studies of the rodent amygdala have led to a neural model of fear conditioning in which the CS+ and US produce signals that arrive in the lateral nucleus and converge there or in the basal nuclear group [26,127]. The resulting signal is transmitted to the central nucleus, which projects primarily to the hypothalamus and midbrain [127,128,129,130]. When this convergence leads to conditioning, the CS+ evokes conditioned responses (CRs) that are evoked by the US, but not by the CS+ before conditioning. Recordings from these structures during surgery for epilepsy demonstrate that the amygdala and frontal lobe structures are activated and interact with each other during fear conditioning [83,131,132,133]. 

Behavioral and electrophysiological data are used to predict components involved in the expectation the painful laser based upon convergent activations by visual and laser stimuli, and correlation with CRs of CS+ SCR or CS+ laser association (Figure 2B and Figure 3, amygdala, and Hippocampal formation composed of HIPP, para hippocampal gyrus, and subiculum). Based upon these data, we have identified scalp EEG activations as sensors for the plasticity of conditioning. Although circuits for aversive conditioning are well known in rodents, the chasm between rodents and humans is large as most cortex in the latter does not exist in the former [27,127,128,134], while the association cortexes that may be found in both differ in the anatomy of projections to amygdala [27,127,128,134,135,136]. Overall, widespread EEG activations may be sensors for cognitive, personality, and emotional behaviors that are important dimensions of fear and anxiety [26,93]. One well-established example for the plasticity of aversive conditioning across species is often related to theta EEG activity (4-7 Hz) [12,29,137]. 

### 3.4. Results: ERSP and CRs

**Figure 4:**
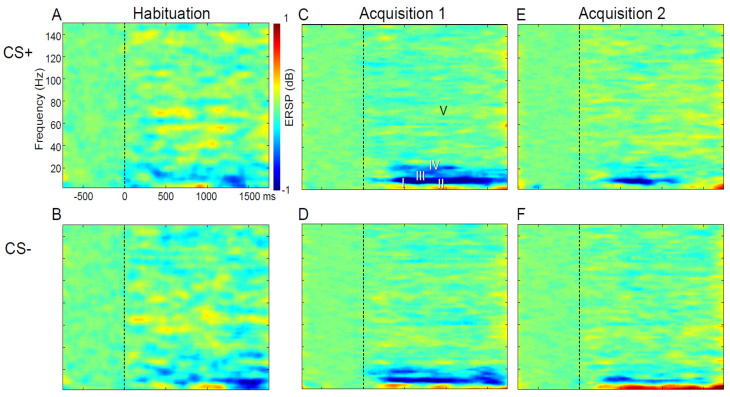
Time Frequency plots of ERSP in (**D**,**B**) across Channels and subjects. CS− and CS+ are shown in the lower and upper rows, respectively. The three columns denote the stage of fear conditioning as labelled. The Habituation stage corresponds to Visual stage in Figure 1 and there are two sequential intervals for the Acquisition stage, i.e., as labelled Acquisition 1 (**C**,**D**) and Acquisition 2 (**E**,**F**) (Figure 1 Legend). Units for the axes of the time frequency plot are shown in (**A**) where time is shown relative to the CS at 0. Windows from I to V are presented in Table 1 (lower 3 rows) and illustrated in (**C**). Within these plots, hot and cold colors show significant increases and decreases in ERSP from that expected at random (green). Adapted from [12].

In humans, components participating in the expectation of pain show EEG activation (ERS/ERD) in fear-related structures at frequencies from the delta to the gamma band (Figure 4, see Abbreviations 1) [12]. Those participating in pain processes per se show pain-related activation by the presence of LEP or ERS/ERD [83,138,139,140]. Both ratings and EEG (LEP and ERS/ERD) might be used as sensors for strength of convergence, and to predict and monitor the effect of therapies for anxiety disorders [141,142].

The human forebrain structures involved in fear conditioning are well characterized by fMRI studies [72]. The amygdala and hippocampus show a contrast of CS+ vs. CS− related EEG and BOLD signals during the interval between the CS and the US in a trace protocol that requires a “memory trace.” In this protocol, the analysis of ERS/ERD can be carried out in the interval between the CS+ and US as an indicator or sensor for anticipation of pain. This is distinct from the interval following the US, which is related to the experience of cued pain (Figure 1). The contrast of CS+ vs. CS− related BOLD signals is related to the SCR, an autonomic expression of the conditioned fear of tolerable painful or “annoying” non-painful electric shock [76,78,143] and loud auditory USs [108]. We used a trace protocol in which the gap between the end of the CS+ and the beginning of the laser US [12,80] is an interval for isolated analysis of anticipation by EEG. Our results so far demonstrate that this protocol leads to behavioral and EEG sensors that may lead to objective biomarkers for fear and anxiety. These results suggest that human cortical structures might contribute to an unpleasant, attentional state of activation during the anticipation of pain, as suggested the relationship between ERSP and ERPs and valence and salience [144,145,146,147,148,149,150]. Cortical contrasts between CS+ and CS− occur in response to CSs at cortical sensory areas that are appropriate for the modality of somatic, auditory, and simple or complex visual CSs [79,110,151].

These results suggest that ratings of CS salience and valence are related to cortical structures giving rise to ERSP and ERPs [12]. In addition, our unpublished findings show that the properties and intensity of the experience of cued pain are correlated with the US SCR and to CS SCR, but not to CS salience or valence. Therefore, the CRs of the CS and US are separate and can be described by analog rather than binary functions, which may increase the resolution of our protocol as a sensor for fear and anxiety. 

The two domains of conditioned fear are consistent with evidence that symptoms in anxiety disorders are related separately to the experience of fearful stimuli and the propensity of an individual to become fearful in response to these stimuli [152]. For example, a large or nearby spider may evoke greater behavioral and fMRI signals than one that is small. In addition, these responses also depend upon individual differences in the tendency to be fearful in general (e.g., neuroticism) or in response to a particular stimulus such as a spider [153,154]. Our protocol could characterize and classify subjects into separate groups, one chiefly dependent on the conditioning stimulus and the other on individual differences in a bottom-up vs. top-down dichotomy [155,156] like that used to model the response to painful stimuli [53]. This model mirrors our experience vs. anticipation classification for fear conditioning with painful stimuli and may have implications for the diagnosis, measurement, and management of anxiety disorders.

### 3.5. Results: ERSP Correlated with Behavior

**Figure 5:**
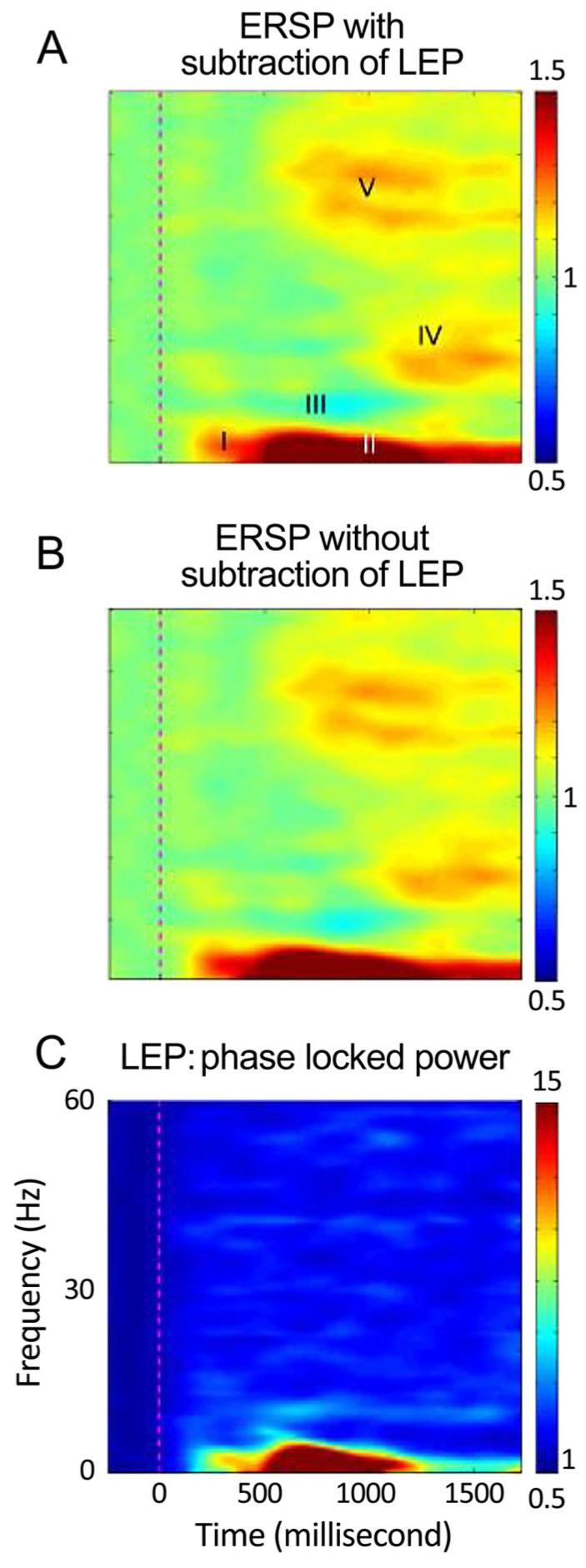
Time-frequency plots of activity in response to the laser pulse. Upper image (**A**): ERS/ERD induced by the laser during the attend laser task averaged across all subjects and channels with subtraction of phase-locked activity. The middle image (**B**) is without subtraction; while the lowest image (**C**) is the plot for ERP evoked by for the LEP. Other conventions as in Figure 4. Adapted from [88].

EEG activities induced by CSs have high temporal resolution that can be used to measure the timing of emotional responses [59] and cortical processes [106,157,158,159] The ERSP may consist of a decrease in the ratio of EEG spectral power over baseline of the EEG (ERD) or an increase in this ratio (ERS). Our studies show that the ERSP following conditioning stimuli can be related to the behavioral response to those stimuli (Figure 2B,C). Figure 4 shows time-frequency plots of ERSP averaged overall relative to the onset of the CS+ and CS− (in rows) for stages of conditioning (Habituation—Hab, Acquisiti on—Acq intervals 1 and Acq 2, in columns, Figure 1 Legend). For each window, the time and frequency ranges (Table 1) are selected to include ERS and ERD components, which are shown in Figure 4 and Figure 5 as ensemble averages of the results across EEG electrodes and subjects. 

ERD characterized WIII and WIV windows (Table 1), and ERS was found in the WV, WI, and WII windows. The WI and WII components were greater for CS− than CS+ in Acq 1 greater than Acq 2 and Hab. WI and WII were most pronounced at prefrontal, frontal, and midline electrodes. WIII and WIV ERD components were larger at occipital and parietal electrodes.

The WV ERS was higher in acquisition than habituation over a broad area from frontal to occipital electrodes. The valence and salience were larger for CS+ than CS−and were correlated with each other and with ERD often at the same electrodes, particularly in WIII. Expectancy and CS SCR were greater for CS+ than CS− (Figure 2A) and were correlated with ERSP at fewer electrodes than valence or salience, which were often correlated with ERSP in WIII. 

These results suggest that ERSP activity induced by the CSs, particularly in WIII, during conditioned fear and reflects cognitive processes such as valence and salience was more than the cognitive and emotional processes expectancy and SCR. Electrodes possibly related to cortical structures seem to mediate both valence and salience, while different cortical (midline) structures and medial temporal structures mediate autonomic responses that reflect fear [29]. In Figure 4, ERSP before and after both the both conditioning stimuli showed delta and theta frequency ERS characterized by earlier and later windows (Figure 4 upper row, WI and WII, Table 1), particularly for CS− vs. CS+. Both the CS+ and CS− showed a WIII ERD component and WIV ERD. Window V was found across the gamma band and the duration of the post-stimulus interval (Table 1). The latencies of these CS induced non-phase-locked ERSP components were consistent with other studies of visual stimuli [160]. 

As mentioned above, self-report metrics for measuring fear and anxiety are indirect since they typically do not include stimuli or environments related to anxiety. These tests are indirect but objective since the patient may not be aware of their performance, and include the Stroop Color-Word test and the dot-probe test measure errors and reaction times during presentation of words or images that are either neutral or threat-related, such as snakes or spiders. There is a broad range of rating 

Scales and questionnaires available to measure fear and anxiety as described in the introduction. Questionnaires provide indirect measures of anxiety and anxiety disorders since they do not include stimuli or environments that are associated with fear or anxiety. Other indirect tests include the Stroop Color-Word Test for words and the dot probe task for words or images that are threatening e.g., snakes or spiders [161,162].

### 3.6. Results: ERSP Correlated with Painful Laser

Human pain mechanisms have been clarified by literature, which shows that pain is a complex experience consisting of several dimensions and their constituent factors: (i) a sensory dimension with factors like the location and intensity of pain, (ii) a cognitive dimension with factors like attention, learning, and declarative memory, and (iii) an emotional dimension with factors of unpleasantness and fear of pain. The first dimension may reflect a bottom-up process while the second and third reflect top-down processes similar to the dichotomy in our model of anxiety as described above [53,152,163]. 

These behavioral dimensions are associated with pain-related activation of cortical and subcortical structures and their component parts [53,149,164,165,166] (see Abbreviations). The fMRI literature and our LFP studies of activation and connectivity [72,83,131,132,133] have shown that these structures constitute a human Pain Network composed of components within one structure, or within adjacent structures to form Local Networks. Both of these types of structures can be included in distributed (hierarchical) networks [167,168,169]. 

Our recent studies have shown that the response to the laser stimulus was characterized by windows (Table 1 lower) in the time-frequency plots (Figure 4 and Figure 5) [88]. The non-phase-locked responses to non-painful electro-cutaneous stimuli were also studied and found to be different from those to painful cutaneous laser stimuli when the baseline salience of the two stimuli is the same, and both of the stimuli are presented at random in a single train. Attention was directed by a task of counting laser vs. non-painful electric stimuli in separate trains where both stimuli are presented randomly in a single train.

Overall, the main effect for Modality (laser vs. electrical) was found for Windows I—delta/theta and V—gamma, and the Interaction of Modality with Task was found for all five windows. Task (attend laser vs. attend electrical stimulus) was found to be a main effect for Window II—delta/theta and Task with Modality interactions were found for all windows. Channel was a main effect for Windows I—delta/theta, II—delta/theta, and III—alpha, and there were no interactions of channel with Modality or Task. However, interactions of Channel with Task and Modality interactions were found for Windows I—delta/theta and III—alpha, and all interaction terms included Modality as a factor. Therefore, the Modality dependent main effects, and the interactions including Modality were the most common effects found in this study and may be generalizable since they were consistent across healthy subjects.

Several studies have examined the effect of directed attention in an oddball protocol upon magneto-encephalographic (MEG) sources activated by a painful intra-cutaneous electrical stimulus. When painful stimuli were counted, an alpha MEG component was found [170], see also [171], which was consistent with our Window III—alpha ERD (Figure 4, top row). Early gamma magnetic components (present window V—gamma) was greater with directed attention to the high intensity target (oddball) stimulus [172]. Overall, these results suggest that attention to painful stimulus induces magnetic components in the alpha and bands ERS, which may be consistent with the present results. 

Linear regression of ERS/ERD for parietal channels (P3, P4, Pz) was most commonly found for sensory (pain or unpleasantness) or attention (salience)-related measures. For parietal channels across several windows, sensory (pain or unpleasantness) or attention (salience) ratings most commonly had significant linear regression on ERS/ERD. These results suggest that a sensor based on alpha ERSP in response to a laser pulse may measure bottom-up processes during attention or vigilance to the painful stimulus that corresponds to the US in our fear conditioning protocol [12,88,173]. 

### 3.7. Results: ERSP Correlated with CRs

We observed that the correlation of salience with ERSP induced by the CSs occurs in the same channels as the correlation of valence with ERSP, and that the ratings of salience and valence are highly correlated across subjects. Some of these channels were located over the cortical structures related to the modality of the CSs. The channels that are correlated with valence or salience are much more common than, and do not overlap with, those for CS SCR or expectancy. This suggests that cortical activity reflects cognitive aspects of the conditioning stimuli rather than expectation or fear. 

The CS salience and valence are correlated with each other and measure the ability of the CS to capture attention, and to express the unpleasantness of the US that may motivate behavior. In this protocol, salience may reflect the attention produced by a threat (CS+) of the painful US. Therefore, the negative valence of the US might account for the relationship between CS valence and salience [150,174,175,176,177]. These properties of visual stimuli are features of the CR to a threat (CS+) and contribute to the unpleasant attentional behavioral state that may define the mood or emotion produced by a threat. 

EEG activity in humans during fear conditioning has been carried out in patients with PTSD vs. controls by using neutral visual CSs, with a trauma reminder as the US [45]. Patients with PTSD showed greater responses to a trauma reminder associated with the CS+ than healthy controls and trauma exposed subjects without PTSD. CRs included SCR and heart rate, as well as increased neural activity (P300), which was an EEG ERP reflecting novelty and calculated as the response to the CS+ minus that to the CS−. The relevance of the P300 to WIII ERD is suggested by its latency and by the effect of task on non-phase-locked frontal central alpha [178] and on gamma oscillatory power [179]. The P300 was recorded over central electrodes during acquisition, again suggesting that the cortical alpha component was related to salience [45,180]. We did not show a difference in alpha ERSP between the CS+ and CS−, consistent with the results of the study above in healthy subjects [45]. 

EEG activity during fear produced by faces showing fear or anger was associated with a frontal theta to beta ratio, which was correlated with subjective measures of attentional control [42]. This theta band activity may correspond to the decrease in Window I and II delta/theta ERS related to threat (CS+ vs. CS−), while Window I ERS was correlated with salience. In particular, these results are consistent with our findings that frontal and prefrontal activity in Windows I and II are greater in response CS− than CS+ and could be related to salience. 

Our results also demonstrate that fear conditioning produces expectancy and consistent increases in alpha ERD (WIII) during acquisition vs. habituation. This expectancy might be reflected in the contingent negative variation, an ERP that is later than the P300 and reflects the learned association between the US and the CS+ [45,181,182]. The contingent negative variation is associated with a decrease in alpha power, which seems to be inconsistent with the decrease in our Window III, because this Window is alpha band ERD, which is a decrease in the ratio of CS induced power over baseline [183,184,185].

Late ERPs and alpha ERD during conditioning or attention can be associated stimuli that are used as CSs whether paired or unpaired with the US [45,182,186], and by laser pulses [82,88,187]. Conditioning of the painful laser US by pairing with the CS+ is correlated with ratings of US SCR, valence and salience [12,53,88], and expectancy, but not with those of CS valence or salience. This suggests that the unpleasant, attentional arousal and neural activity [12,88] associated with the experience of cued pain are related more to pain than to the cue, i.e., the CS+ [24]. These results are consistent with separate domains of anticipation and experience, and show the possibilities of measuring ratings and neural activity as sensors for top-down vs. bottom-up processes as mediators for anxiety and fear [155,156]. 

## 4. Sensors for Fear and Anxiety 

We have adopted the methods of both anticipation and fear by using a formal aversive conditioning protocol with a painful laser US and with measurement of SCR, EEG, and rating scales (Figure 1 and Figure 2) [85,86]. The feasibility of this protocol is shown by CRs that are greater for the CS+ vs. CS− in the case of both the SCR [29,188] and the ratings for CS+ laser association, valence, and salience (inset Figure 2) [12]. This fear conditioning protocol produces a broad range of electrophysiological variables that correlate with CRs and pain related to the laser US. These variables include LEPs, other late potentials like the P300, and non-phase-locked ERS/ERD activity, which may be generalizable based upon their consistency among healthy subjects. In addition, ratings of conditioning or pain during our controlled protocol and are directly related to the fear produced by conditioning. Both types of measure are related to fear and to trait and clinical anxiety, and both can change over short time intervals like those for some moods and emotions [57,58,59]. These measures could also be useful for diagnosis and assessment of treatment response of anxiety and anxiety disorders [189,190]. The feasibility of this type of instrumented test is suggested by standard neuropsychological tests that produce objective measures of sustained visual attention and vigilance to visual modalities [191,192,193,194]. A congruent test for fear conditioning could produce sensors for multiple aspects of fear, including autonomic and cognitive behavioral measures. 

These protocol-related measures correspond to separate behavioral domains of conditioned fear anticipation as manifested by CRs and experience by changes in the dimensions of pain. The former is the anticipation of pain produced by the CS [12,116] that are associated with CRs, including: (i) CS SCR and (ii) ratings of CS+ laser association, salience, and valence [12,24,25]. The CS+ laser association is be measured by a scale on which the subject may not be aware of the association. Scales of this type are particularly important because they measure fear or expectancy directly and objectively [35,36].

The other domain is that the experience of cued pain is produced by the US and is associated with CRs of US SCR and pain that are related to CS SCR but not CS salience or valence, which supports the model of two separate domains of conditioned fear. The anticipation domain may result in fear and avoidance of pain whereas the experience domain may facilitate the escape from pain, as two separate aspects of the fear of pain [12,195]. Behavioral and EEG measures for both domains are indirect and objective, and are segmented into a range of psychological dimensions, some of which may not engage awareness [35,36]. The potential sensors based on EEG techniques have some advantages over fMRI-based sensors, which measure neural activity indirectly and at much lower temporal resolution [52,53,54]. However, MRI has the advantage that it surveys the whole brain while scalp EEG surveys multiple cortical and subcortical structures that cannot be specified but only estimated at low spatial resolution by source analysis [196,197]. Combinations of both types of measures may exploit the strengths of each and produce valuable biomarkers. 

The results described above could be used to assess the potential of this protocol as an objective instrumented test to measure fear and anxiety. The extent to which experimental fear (CS+ SCR, CS+ laser association) and salience are related to clinical fears could initially be assayed by the questionnaires listed in the introduction. Thereafter, a canonical correlation analysis could be used to examine the relationship between the experimental and clinical measures of fear. Further, a coefficient measuring the strength of association between the two multivariate measures could be computed produce a weighted sum of both, which would provide latent summary measures for each variable. If experimental fear is related to the clinical fear, then predictive models for this relationship might be generated using machine learning techniques such as clinical fear and anxiety from tests for experimental fear, as in the approach to models for pain prediction [198]. The clinical utility of these models could then be assessed by studies of patients with anxiety disorders, focusing first on panic disorder. Conditioned fear is most evident in these patients and there is evidence that treatment response is related to CNS activity during conditioning [189,190]. 

The clinical utility of instrumented tests for fear and anxiety is evident in some situations encountered during subjective assessment of symptoms for diagnosis, treatment, decision making, and monitoring of treatment response over time. For most psychiatric disorders, there is no gold standard method for clinical assessment, which is often very complicated. One example is when the lack of a treatment response leads the clinician to question simultaneously both the diagnosis and treatment options [199]. In addition, reliance on subjective assessment may place the clinician in the difficult situation of deciding whether the patient is reporting symptoms accurately or exaggerating the type or severity of symptoms for financial benefits or other secondary gains [200,201]. This confound is well established in PTSD when there is a possibility of compensation from the veterans administration, and in other diagnoses, such as chronic pain following injuries on the job [20,202,203]. Conversely, patients (e.g., active duty soldiers) may minimize their symptoms because of concern about losing their livelihood and identity as warriors [204]. Given these limitations of purely subjective assessments of anxiety, objective measures may provide useful additional evidence to the overall the clinical picture, as they have in the diagnosis and management of disorders of attention such as Attention Deficit Hyperactivity Disorder and Traumatic Brain Injury [205]. 

## Figures and Tables

**Table 1:** Latency and dimensions of the frequency band for Windows of ERSP components, either ERS or ERD, following the laser in the Attend Laser Task and the conditioning stimuli in the fear conditioning protocol.

	ERSP Window I (WI), Delta/Theta	ERSP Window II (WII), Delta/Theta	Window III (WIII), Alpha	Window IV (WIV), Beta	Window V (WV) Gamma
**Attend Laser Protocol: Latency is Time after the Laser Pulse**					
ERSP induced by Painful Laser US	ERS	ERS	ERD	ERS	ERS
Latency	200–400 ms	600–1400 ms	500–900 ms	1200–1600 ms	800–1200 ms
Frequency	0–8 Hz	0–8 Hz	8–10 Hz	15–25 Hz	40–50 Hz
**Fear Conditioning protocol: Latency is Time after the CS**					
Conditioning stimuli—CS ERS/ERD	ERS	ERS	ERD	ERD	ERD/ERS
Latency	190–500 ms	600–1400 ms	200–1400 ms	200–1600 ms	0–1200 ms
Frequency Range	0–8 Hz	0–8 Hz	8–14 Hz	16–25 Hz	30–120 Hz
